# *In vitro* untargeted polar metabolomics data from *B. cenocepacia* and *S. aureus* biofilm supernatants

**DOI:** 10.1128/mra.00261-25

**Published:** 2025-05-07

**Authors:** Hayden Skaggs, Deborah R. Yoder-Himes

**Affiliations:** 1 Department of Biology, University of Louisville 5170, Louisville, Kentucky, USA; Rochester Institute of Technology, Rochester, New York, USA

**Keywords:** *Burkholderia*, *Staphylococcus*, biofilm, metabolome, supernatant

## Abstract

Here, we offer a metabolomics dataset generated via high-performance liquid chromatography and high-resolution mass spectrometry analysis of *in vitro* biofilm supernatant harvested from the human pathogens *Burkholderia cenocepacia* and *Staphylococcus aureus*. A total of 618 polar metabolites were identified across all experimental groups.

## ANNOUNCEMENT

*Staphylococcus aureus* and *Burkholderia cenocepacia* are well-studied pathogens (1–4) that can colonize individuals who are immunocompromised or with the genetic condition cystic fibrosis (CF) (5). The polymicrobial nature of the CF lung makes it difficult to obtain distinct *in vivo* metabolic profiles for specific pathogens (6–8). Further, freely accessible metabolomics data sets for bacteria, particularly in non-planktonic conditions, remain paltry compared with eukaryotes. This study aims to identify the extracellular metabolites produced by these pathogens under biofilm conditions in order to better understand the complex metabolome of the CF lung.

Collection of cell-free supernatants from epidemic isolates of *B. cenocepacia*, H111 and J2315, and *S. aureus* NRS77 from quadruplicate 7-day-old mono-culture biofilms was done as previously described (9). The sterile supernatant from each of the three cultures and the uninoculated medium (LB Lennox broth + 150 mM MOPS + 1% glucose) was lyophilized using a LabConco FreeZone 2.5L benchtop freeze dryer for 24 h. Dried supernatants were reconstituted in 2 mL 50% acetonitrile and injected into a Thermo DIONEX UltiMate 3000 HPLC system, then analyzed using a Thermo Q Exactive HF Hybrid Quadrupole-Orbitrap Mass Spectrometer. The LC system was equipped with a reversed phase column (Waters Acquity UPLC HSS T3 column, 2.1 × 150 mm, 1.8 µm) and hydrophilic interaction chromatography column (Millipore SeQuant ZIC-cHILIC column, 2.1 × 150 mm, 3 µm). Information regarding specific column conditions and solvent gradient can be found in our related work (6). To identify metabolites, 2D LC-MS/MS data were first matched to our own proprietary database that contains parent ion m/z, MS/MS spectra, and retention time of 363 authentic standards. Thresholds were set as spectral similarity ≥0.4, retention time difference ≤0.15, and m/z variation window ≤5 ppm. 2D LC-MS/MS data without a match with the metabolites in the proprietary database were further analyzed using Compound Discoverer software (v 3.1, Thermo Fisher Scientific, Germany) as described in (9). A total of 618 polar metabolites were identified between the three culture supernatants and the uninoculated culture media. Principal component analysis (PCA) was performed using Origin(pro) graphing software v2024b. For all software listed above, default parameters were used unless otherwise noted.

**Fig 1:**
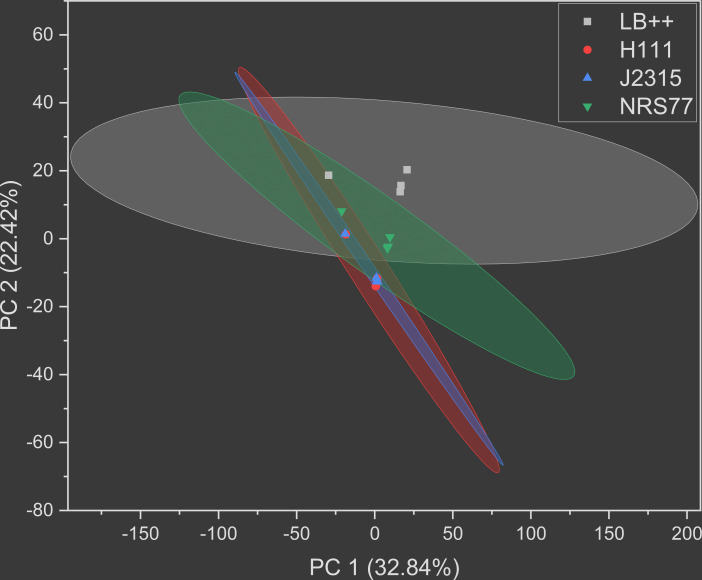
Principal component analysis (PCA) scores plot of biofilm supernatant metabolic profiles. Principal component analysis was performed on the raw chromatographic peak intensities of each metabolite identified across all experimental conditions. One data point represents a single biological replicate. LB++ (gray), *B. cenocepacia* H111 (red), *B. cenocepacia* J2315 (blue), and *S. aureus* NRS77 (green). Sterile supernatant of uninoculated LB + 1% glucose + 150 mM MOPS (LB++) serves as the control condition, with all further samples being characterized in this same media. The axes represent the percentage of variance for that respective principal component. The colored ellipses represent the 95% CI of PCA scores. Samples with similar scores will appear close to each other.

Principal component analysis (PCA) was performed on the raw chromatographic peak intensities for each of the metabolomic data sets. The PCA scores plot depicts strong similarity between the metabolic profiles of the *B. cenocepacia* clinical isolates in the form of overlapping 95% confidence interval (CI) ellipticals (Fig. 1). The *S. aureus* laboratory strain exhibits a slight similarity in its metabolic profile with the *Burkholderia*, but is otherwise independent. The control condition was as expected.

Overall, the untargeted polar metabolomics data set provides a basis for understanding the extracellular biofilm metabolome of these pathogens. Identifying the metabolic secretions of these organisms is paramount to understanding their pathogenicity within the CF lung. These data could be used to explore differences between *in vitro* and *in vivo* CF metabolomes, other microbial interactions in the CF lung, interactions between these pathogens and the human host immune systems, and in a broader sense, other polymicrobial infections.

## Data Availability

The full metabolic dataset for each sample identified in the study, including the raw chromatographic peak intensities for each metabolite identified, has been made publicly available in figshare at the following link: https://doi.org/10.6084/m9.figshare.28543700.v1.
